# Mapping the immune environment: spatiotemporal dynamics in cardiovascular events

**DOI:** 10.3389/fimmu.2026.1809941

**Published:** 2026-04-23

**Authors:** Rasit Dinc, Nurittin Ardic

**Affiliations:** 1 INVAMED Medical Innovation Institute, New York, NY, United States; 2 Med-International UK Health Agency Ltd., Nuneaton, Warwickshire, United Kingdom

**Keywords:** cardiovascular inflammation, immune compartments, molecular imaging, precision immunomodulation, spatial transcriptomics, spatiotemporal dynamics

## Abstract

Cardiovascular disease remains a leading cause of global mortality despite advances in revascularization, pharmacotherapy, and risk stratification. Immune responses to cardiovascular damage are neither temporally linear nor spatially homogeneous; they evolve across overlapping phases and distinct anatomical compartments. Here, we propose a four-dimensional spatiotemporal framework to map immune dynamics in cardiovascular events. Temporally, responses progress from hyperacute thrombo-inflammatory activation to acute inflammation, resolution/repair, and chronic remodeling. Spatially, immune programs differentiate across the intravascular compartment, infarct/lesion core, peri-infarct border zone, distant myocardium, and device-tissue interface, each possessing distinct cellular composition, signaling pathways, and therapeutic sensitivities. By integrating molecular imaging, single-cell and spatial transcriptomics, serial biomarker profiling, and computational modeling, we demonstrate how immune activity can be resolved with increasing anatomical and temporal precision. Using neutrophil extracellular traps as a case study, we show how the same effector mechanism can exhibit different effects depending on the phase and microenvironment. Clinical trial experience with anti-inflammatory therapies further emphasizes the need to align target selection and treatment timing with the underlying immunological trajectories. A spatiotemporal approach transforms cardiovascular inflammation from a static risk factor into a measurable and potentially targetable dynamic, site-specific process. Including phase, anatomical niche, and immune phenotype in experimental design and clinical interpretation can improve precise immunomodulation and strengthen translational alignment in cardiovascular medicine.

## Graphical Abstract

**Figure:**
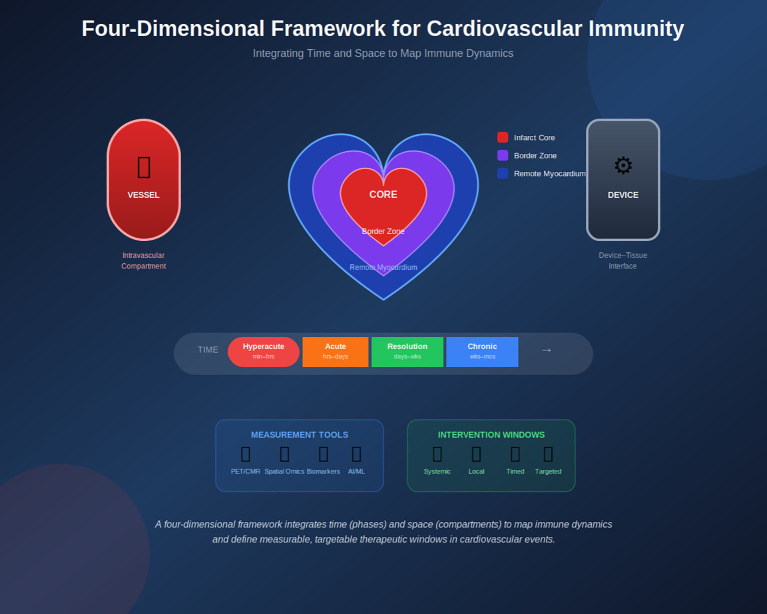

## 1 Introduction

Cardiovascular events are immunological processes that unfold across space and time. Acute myocardial infarction (MI) begins within seconds, with platelet activation and leukocyte recruitment, yet its immunological consequences may persist for months through cycles of inflammation, resolution, and remodeling (1, 2). At the population level, cardiovascular disease remains the leading cause of death worldwide, highlighting the importance of improving mechanistic frameworks that can better inform preventive and therapeutic strategies (3, 4). Despite decades of research on immune mediators in cardiovascular injury, a key gap remains: we often know what occurs, but not where or when. The dominant paradigm still treats cardiovascular inflammation as temporally limited but spatially homogeneous. Reviews catalog immune mediators; clinical trials frequently test anti-inflammatory agents at fixed time points; and biomarker studies often measure circulating factors without establishing a clear link to their tissue origins or anatomical contexts (1, 5). This approach has produced significant advances, including evidence that targeted anti-inflammatory strategies can reduce cardiovascular events in selected settings (6). Colchicine has also shown benefit in secondary prevention populations, reinforcing the translational importance of inflammatory pathways (7, 8). However, broader experience has included inconsistent results across candidate pathways and populations, likely reflecting a lack of congruence between intervention timing, anatomical targeting, and immune phase-specific biology (2, 5).

Although the framework presented here is generally applicable to cardiovascular inflammatory conditions, this review focuses primarily on the immune dynamics that arise following acute MI and subsequent progression toward left ventricular remodeling and chronic ischemic heart failure. MI represents the best-defined clinical context in which spatially and temporally organized immune responses determine structural remodeling and long-term cardiac function. Thus, MI serves as a representative model to demonstrate how immune mechanisms evolve across anatomical sections and temporal phases.

Immune responses differ significantly across anatomically distinct cardiovascular compartments. The infarct core, shaped by necrosis, hypoxia, and dense early myeloid infiltration, creates a unique microenvironment capable of reprogramming immune phenotypes and effector functions (1, 9). The peri-infarct border region, where viable myocardium interfaces with damaged tissue, integrates inflammatory and reparative signals and can critically influence infarct expansion and subsequent ventricular remodeling (2, 9). Distant myocardium, once considered immunologically immobile, is increasingly recognized as susceptible to systemic immune overflow and neuroimmune signaling, leading to potential consequences for adverse remodeling trajectories (10). Beyond native tissue, device-tissue interfaces reveal biomaterial-specific immune kinetics, foreign body responses, and thrombo-inflammatory cascades that influence post-implantation outcomes (11, 12).

The temporal dimension is equally critical. The immune response to cardiovascular injury progresses through overlapping phases, including a hyperacute window of intravascular activation (minutes to hours); an acute inflammatory phase with vigorous myeloid uptake (hours to days); a resolution/repair phase characterized by efferocytosis and macrophage reprogramming (days to weeks); and a chronic remodeling phase (weeks to months). In this stage, persistent inflammation can lead to fibrosis and progression of heart failure (1, 2, 13). Interventions that are beneficial in one stage may be neutral or harmful in another, highlighting that timing is not a practical detail but a fundamental biological variable (2).

The technology for mapping cardiovascular immunity in four dimensions (three spatial and one temporal) is now mature. Single-cell and spatially resolved transcriptomic approaches can identify immune and stromal programs *in situ* and correlate them with anatomical niches (9, 14). Molecular imaging, including inflammation-sensitive positron emission tomography (PET) strategies, enables non-invasive monitoring of immune activity and bridges the gap between mechanistic insight and clinical phenotyping (10). Serial biomarker profiling and computational approaches can reconstruct immune trajectories from accessible samples, and machine learning methods are becoming increasingly capable of integrating high-dimensional cardiovascular data to identify patterns not apparent with conventional analysis (15).

In this review, we propose a four-dimensional spatiotemporal framework to conceptualize immune dynamics in cardiovascular events. Instead of cataloging mediators or summarizing disease-specific findings in isolation, we present a structured architecture that integrates temporal immune phases (hyperacute, acute, resolution/repair, chronic remodeling) with spatial compartments (intravascular, infarct/lesion core, border zone, distant myocardium, and device-tissue interface). This phase-compartment matrix facilitates systematic analysis of how immune populations localize and evolve, with direct implications for biomarker interpretation, therapeutic targeting, and clinical trial design. By clearly organizing current evidence temporally and spatially, we aim to clarify why immune interventions succeed or fail, identify viable therapeutic windows, and outline future directions for real-time immune monitoring and precise immunomodulation in cardiovascular care.

### 1.1 Positioning of the present review

Several influential reviews exist that define immune mechanisms in MI and cardiac remodeling (1, 9, 16). These studies primarily focus on cellular mediators and molecular pathways driving post-infarction inflammation and repair. The present review differs in its conceptual organization, proposing a structured spatiotemporal framework that integrates the immune phase (hyperacute, acute inflammation, resolution/repair, and chronic remodeling) with defined anatomical segments (intravascular, infarct core, border zone, distant myocardium, and device-tissue interface). By combining temporal immune phases with spatial segmentation, the framework provides a systematic architecture for interpreting biomarker dynamics, imaging signals, and therapeutic intervention windows.

## 2 Temporal architecture of cardiovascular immune response

The immune response to cardiovascular injury forms an overlapping temporal continuum. Although often summarized as “repair following inflammation,” the post-injury response can be better understood as a series of phase-dominant programs, each with its own characteristic cellular composition, effector functions, and regulatory checkpoints. The biological role of inflammation changes over time: the same pathway may be protective (clearance and containment) in the early stages, but maladaptive (fibrosis, dilation, heart failure) later (1, 2). A temporally explicit framework is essential for interpreting biomarkers, understanding clinical trial outcomes, and defining therapeutic windows.

### 2.1 Hyperacute phase (minutes to hours)

Within minutes of ischemia, plaque rupture, or device-related vascular injury, the intravascular space becomes a dominant immunological field. Platelet activation and endothelial disruption rapidly promote leukocyte tumbling, adhesion, and platelet-leukocyte aggregate formation. In parallel, neutrophils undergo early activation and NET formation, contributing to immunothrombosis and enhancing local damage at the blood-intima interface (5, 17). NET-induced thrombo-inflammatory amplification is increasingly recognized in arterial and venous thrombosis, supporting NETs as a unifying mechanism of action in acute occlusive events. In patients with ST-segment elevation myocardial infarction (STEMI), the NET burden within coronary thrombi correlates with ST segment resolution and infarct size, supporting a clinically significant hyperacute NET signal in coronary occlusion (18). Clinical biomarker studies also support the coordinated activation of NET-associated markers and complement components in coronary disease, supporting coupled innate effector systems in thrombo-inflammatory risk states (19).

Complement activation is also an early component of myocardial ischemia-reperfusion injury and contributes to endothelial activation, leukocyte recruitment, and microvascular dysfunction. A specific review focused on MI highlights complement effector mechanisms and summarizes previous anti-complement therapeutic concepts in the context of reperfusion (20). Recent human data support the association of dysregulated C5a–C5aR1 signaling with endothelial damage in acute MI and the role of complement as an early vascular amplifier and a potential therapeutic node (21). Hyperacute NET-thrombus dynamics and complement activation together create a short but biologically powerful window in which intravascular inflammation and thrombosis strongly interact and can influence downstream infarction evolution.

### 2.2 Acute inflammatory phase (hours to days)

The acute inflammatory phase is characterized by the rapid accumulation of innate immune cells in the damaged myocardium. Neutrophil infiltration typically increases early and peaks within the first 1–3 days (depending on species/model), contributing to debris clearance but also releasing proteases, reactive oxygen species, and additional NETs that can exacerbate tissue damage and microvascular obstruction (1, 5). Parallel recruitment of monocytes shapes infarct inflammation and sets the stage for subsequent repair. Myeloid cells exhibit functional heterogeneity and plasticity, with early inflammatory macrophage programs supporting clearance and cytokine amplification, while later transitions promote resolution (2, 22).

This acute phase is also the period when cytokine-targeted anti-inflammatory approaches demonstrate clinical benefit in selected populations. For example, inhibition of the IL-1β pathway reduced recurrent cardiovascular events in high-risk patients, highlighting the translational importance of inflammatory signaling and suggesting that patient selection and timing are likely important (6). Acute inflammation is not inherently harmful; it is required for debris clearance and organized repair. Pathological risk arises when the inflammation program is excessive, prolonged, or spatially misdirected (1, 2).

### 2.3 Resolution and repair phase (days to weeks)

Resolution is an active immunological program rather than a passive decline of inflammation. At this stage, macrophage populations undergo functional reprogramming toward reparative phenotypes characterized by increased efferocytosis, release of anti-inflammatory mediators, and regulation of stromal responses that promote wound formation (2, 23). Successful efferocytosis, particularly the clearance of apoptotic neutrophils and necrotic debris, terminates inflammatory signaling and promotes fibroblast activation and extracellular matrix accumulation (1, 23). This period is a crucial turning point for long-term outcomes: inadequate healing can perpetuate inflammation, while excessive fibroblast activity can accelerate non-conforming fibrosis. The overlap between decreased inflammation and increased fibrotic repair highlights that indiscriminate immunosuppression late in the process may impair healing, whereas strategies that strengthen healing pathways may be beneficial (2).

### 2.4 Chronic remodeling phase (weeks to months)

After wound stabilization, low-grade immune activity often may contribute to chronic remodeling. In the post-infarction setting, remodeling primarily refers to left ventricular remodeling, characterized by ventricular enlargement, fibrosis, and a progressive decrease in contractile function (2, 24). Late-stage complementary syntheses highlight that overlapping immune-stromal programs during late infarct healing shape long-term remodeling trajectories and therapeutic opportunities (13). Ongoing inflammatory signaling and altered immune-stromal interaction can lead to progression toward interstitial fibrosis, ventricular dilation, and heart failure (1, 2). Systemic immune activation may also retain its importance. Monocyte/macrophage biology in the post-myocardial infarction period has been extensively studied, including sustained inflammatory programs and their association with the risk of heart failure. These demonstrate why non-specific immunosuppression frequently fails and why more precise temporal targeting is needed (23).

Complement signaling may also maintain its mechanistic importance beyond the hyperacute window. Experimental and translational studies point to C5a–C5aR signaling in myocardial ischemia-reperfusion injury and infarction outcomes, suggesting that complement-driven leukocyte-endothelial interactions are part of the inflammatory continuum (25). Clinically, classical patient studies have demonstrated measurable complement activation products (including anaphylatoxins such as C3a and C5a and terminal complement complex) after acute MI, supporting the idea that complement activation is not merely a laboratory observation but also occurs in human diseases (26).

Understanding these temporal dynamics is crucial for designing time-appropriate immunomodulatory interventions and interpreting biomarker trajectories in clinical practice. **Table 1** summarizes the four distinct temporal phases of the cardiovascular immune response following myocardial injury, from the hyperacute to the chronic phase. For each phase, the dominant cellular components, key molecular pathways and mechanisms, representative circulating and tissue biomarkers, clinical correlations, and therapeutic considerations are presented. **Table 1** summarizes the four temporal phases of the cardiovascular immune response, emphasizing their distinct cellular and molecular characteristics.

**Table 1:** Temporal phases of cardiovascular immune response.

Phase	Dominant cellular components	Key pathways/mechanisms	Representative biomarkers	Clinical correlates	Treatment considerations
Hyperacute (minutes–hours)	Platelets, neutrophils, endothelial cells	Immunothrombosis; complement activation; NET formation; DAMP signaling	Platelet-leukocyte aggregates, NET markers (dsDNA, citH3), complement fragments (C3a, C5a)	Coronary occlusion, microvascular obstruction, no-reflow	Antithrombotics; complement inhibition; early anti-inflammatory targeting
Acute inflammatory (hours–days)	Neutrophils; CCR2^+^ monocytes; inflammatory macrophages	IL-1β and TNF signaling; ROS production; cytokine amplification	IL-6, CRP, myeloperoxidase, circulating monocyte subgroups	Infarct expansion, edema, premature remodeling	Targeted cytokine inhibition (e.g., IL-1 axis); colchicine; timing-sensitive modulation
Resolution/repair (days–weeks)	Reparative macrophages; fibroblasts; endothelial cells	Efferocytosis; macrophage reprogramming; extracellular matrix accumulation	Declining CRP; growth factors; matrix turnover markers	Scar formation, infarct stabilization	Promoting healing; avoiding excessive suppression of reparative immunity
Chronic remodeling (weeks–months)	Macrophages; T cells; fibroblasts	Sustained low-grade inflammation; fibrotic signaling; immune-stromal interaction	Persistent inflammatory markers; fibrosis-related peptides	Ventricular dilatation; heart failure progression	Anti-fibrotic strategies; targeted immune modulation; trajectory-based intervention

**Figure 1:**
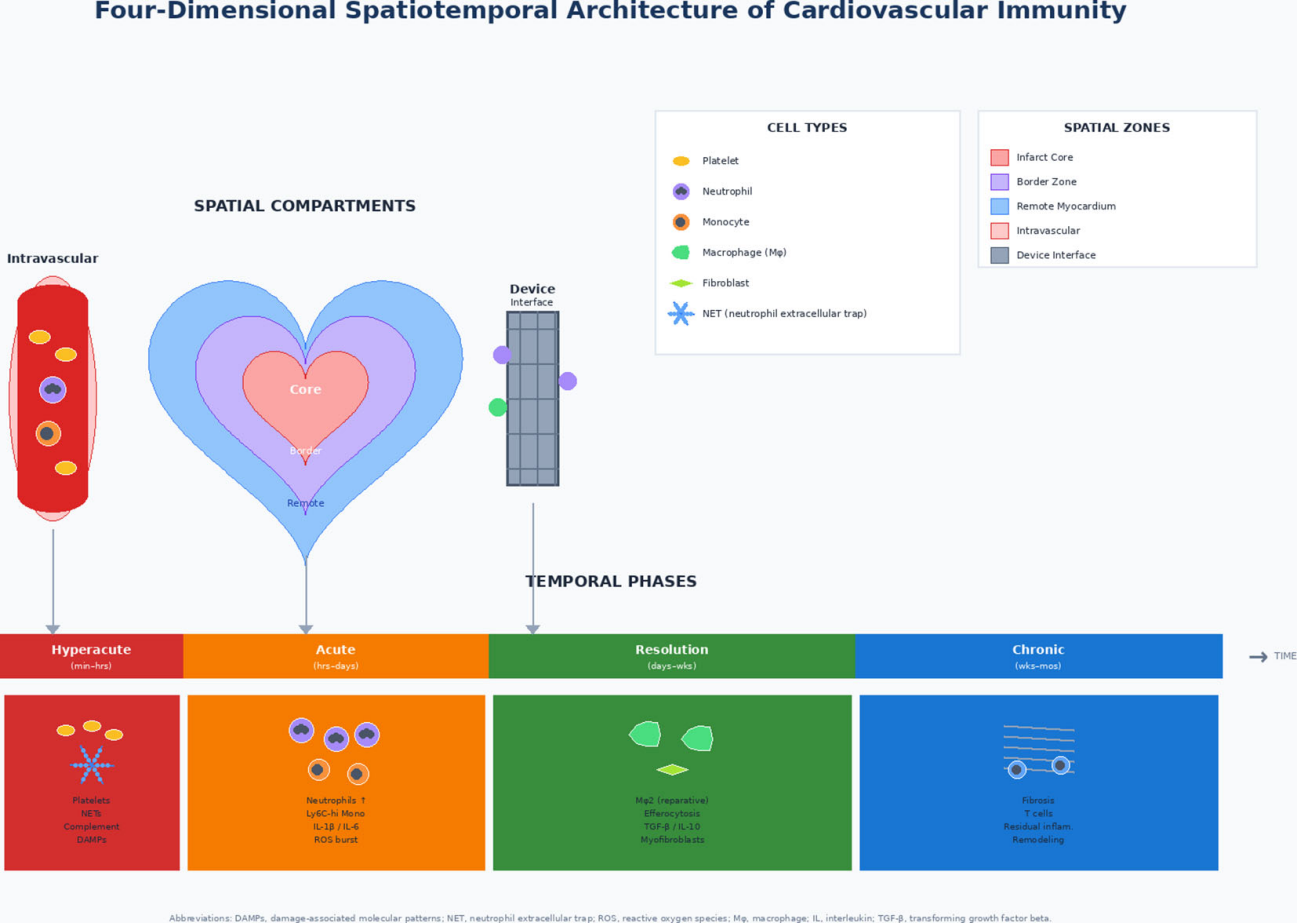
Four-dimensional spatiotemporal architecture of cardiovascular immunity. This schematic illustrates an integrated four-dimensional (4D) framework of cardiovascular immunity, depicting how immune responses following myocardial injury vary across both spatial compartments and temporal phases. Spatial compartments (top panel): The central heart illustration shows three concentric tissue regions: the core (red, innermost), the peri-infarct border zone (purple, center), and the remote myocardium (blue, outermost). The intravascular compartment (left) is shown as a blood vessel containing circulating platelets, neutrophils, and monocytes. The device-tissue interface (right) shows a stent/network structure along with peripheral cellular responses representing the foreign body reaction. Temporal phases (bottom panel): Four color-coded phases range from minutes to months after injury. The hyperacute phase (red) includes platelet activation, NET release, complement activation, and DAMP signaling. The acute inflammation phase (orange) is characterized by neutrophil infiltration, Ly6C-high monocyte recruitment, pro-inflammatory cytokines (IL-1β, IL-6), and ROS production. The resolution/repair phase (green) shows transition to reparative Mφ2 macrophages, efferocytosis, anti-inflammatory mediators (TGF-β, IL-10), and myofibroblast activation. The chronic remodeling phase (blue) shows fibrosis, T cell recruitment, and ongoing tissue remodeling. DAMPs, damage associated molecular patterns; IL, interleukin; Mφ, macrophage; NET, neutrophil extracellular trap; ROS, reactive oxygen species; TGF-β, transforming growth factor beta.

The proposed four-dimensional framework for synthesizing these concepts is schematically shown in **Figure 1**.

## 3 Spatial segmentation of cardiovascular immunity

Temporal phase alone cannot explain the heterogeneity of cardiovascular immune responses. Immune activation occurs within anatomically and microenvironmentally distinct compartments, each characterized by unique cellular composition, metabolic constraints, vascular dynamics, and stromal architecture. A spatiotemporal framework therefore requires defining co-existing immune niches (1, 2).

### 3.1 Intravascular compartment

The intravascular space is an active immunological interface. Following plaque rupture or endothelial damage, platelet activation, von Willebrand factor exposure, and neutrophil recruitment form platelet-leukocyte aggregates that promote immunothrombosis (5, 17). NET components were directly visualized in coronary thrombi of myocardial infarction patients with ST segment elevation, and intraluminal NET load was correlated with infarction size and reperfusion outcomes (18).

Beyond acute thrombosis, circulating immune cell phenotypes and inflammatory mediators serve as dynamic biomarkers of tissue damage. Elevated monocyte subgroups and inflammatory cytokines are associated with a risk of adverse remodeling, suggesting that peripheral immune signatures partly reflect events at the tissue level (23). However, the intravascular space is not simply a mirror of myocardial inflammation; it represents a distinct microenvironment shaped by shear stress, complement activation, and platelet-driven signaling. Therefore, the intravascular niche constitutes both an initiator of tissue damage and a measurable reservoir of biomarkers.

### 3.2 Infarct or lesion core

The infarct core represents an extreme microenvironment metabolically and immunologically, characterized by hypoxia, necrosis, acidosis, and high DAMP concentrations. These conditions trigger intense neutrophil infiltration in the early stages and create a pro-inflammatory cytokine environment (1).

Recent spatially transcriptomic mapping of human myocardial infarction has shown distinct gene expression regions within the infarct tissue and confirmed that immune and stromal programs are not homogeneously distributed but clustered in defined regions (9). These analyses highlight the biological distinctiveness of the infarct core, revealing region-specific activation of inflammatory and extracellular matrix pathways. Time-resolved spatial multi-omics in experimental MI further support the microanatomical organization of inflammatory niches and immune infiltration pathways in the early stages of injury (27).

In this compartment, immune responses are initially dominated by clearance and containment functions. However, excessive proteolytic activity or prolonged inflammatory signaling can destabilize the extracellular matrix and increase the risk of infarct expansion (2).

### 3.3 Borderline or peri-infarct zone

The border region, where viable myocardium interfaces with necrotic tissue, is a critical determinant of long-term ventricular remodeling. Unlike the infarct core, this region contains metabolically active cardiomyocytes exposed to inflammatory mediators, oxidative stress, and varying mechanical stress.

Experimental and translational studies highlight that immune signaling in the border region strongly influences infarct thinning, scar maturation, and adverse remodeling (2). Spatial multiple omics analyses also demonstrate that reparative and inflammatory programs coexist in this region, reflecting dynamic immune-stromal cross-interaction (9).

The border region may constitute a therapeutically viable niche: where modulating immune activity can affect infarct spread without disrupting essential clearance functions in the necrotic core.

### 3.4 Distant myocardium and systemic interaction

Cardiovascular damage triggers systemic immune activation that extends beyond the primary lesion. Distant myocardium, initially undamaged, can contribute to global ventricular remodeling by exhibiting inflammatory cell infiltration and altered gene expression (1). Molecular imaging studies using inflammation-sensitive PET tracers have demonstrated persistent myocardial inflammatory activity distant from the infarction site and revealed that this activity is associated with remodeling trajectories (10).

Systemically, splenic monocyte reserves and bone marrow hematopoiesis participate in post-myocardial infarction immune dynamics. Experimental and clinical studies have highlighted the role of cardiosplenic axis activation in maintaining post-infarction inflammatory cell supply (22). Recent studies further reinforce the concept of the cardio-spleen axis shaping leukocyte supply and prognostic outcomes after heart injury (28). Human autopsy and translational data also strengthen multi-organ immune connectivity beyond the infarct site, suggesting structured spleen immune remodeling after fatal MI (29). This systemic-cardiac connection reinforces the idea that cardiovascular immunity is multi-organ and cannot be limited to the infarct site alone.

### 3.5 Device-tissue interface

Implantable cardiovascular devices, stents, valves, vascular grafts, and mechanical circulatory support systems, introduce additional spatial complexity. The device-tissue interface constitutes an immune niche characterized by biomaterial-dependent foreign body responses, macrophage polarization shifts, and thrombo-inflammatory cascades (11, 12). In vascular interventions, the balance between endothelial healing, smooth muscle proliferation, and local inflammation determines the risk of restenosis. In cardiac implants, persistent low-grade inflammation at the biomaterial interface can affect fibrosis and device integration. Unlike natural infarct tissue, this niche is defined not by necrosis but by biomaterial recognition and mechanistic interaction. New device concepts aim to actively shape peri-device immunity through biologically inspired and biomimetic surface strategies, including vesicle- and exosome-mimicking engineering approaches (30-a). Similarly, biodegradable scaffold platforms exhibit different healing kinetics and immune-stromal responses compared to persistent metallic implants, reinforcing the need to treat the ‘device-tissue interface’ as its own immune compartment (31). The inclusion of the device-tissue interface within a spatiotemporal immune framework extends cardiovascular immunology beyond classical ischemic damage and aligns the mechanistic understanding with contemporary interventional applications.

Recognition of spatial heterogeneity is critical for directing therapeutic interventions to appropriate tissue compartments and selecting optimal sampling strategies in translational research. **Table 2** describes five anatomically and functionally distinct spatial compartments involved in the cardiovascular immune response; each compartment is characterized by unique microenvironmental features, dominant immune processes, key cellular players, preferred measurement methods, and clinical outcomes.

**Table 2:** Spatial segmentation of cardiovascular immunity.

Compartment	Microenvironmental features	Dominant immune processes	Key cellular players	Measurement modalities	Clinical implications
Intravascular compartment	Shear stress; exposed subendothelium; thrombus formation	Platelet-neutrophil interaction; NET-induced thrombosis; complement activation	Platelets; neutrophils; circulating monocytes	PET (vascular); blood biomarkers; flow cytometry	Culprit lesion thrombosis; residual inflammation risk
Infarct/lesion core	Hypoxia; necrosis; acidosis	Neutrophil infiltration; DAMP-induced inflammation; proteolysis	Neutrophils; inflammatory macrophages	Spatial transcriptomics; CMR; USPIO/ferumoxitol imaging	Infarct size; microvascular obstruction
Border/peri-infarct region	Viable cardiomyocytes + inflammatory signals; mechanical strain	Mixed inflammatory-reparative signaling; immune-stromal interaction	Macrophage subtypes; fibroblasts	PET; spatial multi-omics; tissue histology	Infarct expansion; adverse remodeling
Remote myocardium	Systemic immune spillover; neuroimmune signaling	Low-grade inflammation; remodeling signaling	Monocytes; macrophages; T cells	PET imaging; plasma biomarkers	Global ventricular remodeling; HF risk
Device-tissue interface	Biomaterial contact; altered flow; mechanical forces	Foreign body response; macrophage polarization; thromboinflammation	Macrophages; giant cells; neutrophils	Histology; molecular profiling; device surface analysis	Restenosis; fibrosis; device integration outcomes

## 4 Integration of spatiotemporal data: methodological approaches

A spatiotemporal framework for cardiovascular immunity is only as useful as the tools currently available to measure it. Traditional approaches (static histology, single-timepoint plasma biomarkers, or bulk transcriptomics) cannot fully resolve site-specific heterogeneity or reconstruct immune trajectories over time. Recent advances allow for clear longitudinal and spatial investigation of immune activity across the myocardium, vascular system, and peripheral immune system.

### 4.1 Molecular imaging of cardiovascular inflammation

Non-invasive molecular imaging can localize *in vivo* inflammatory activity and enable serial monitoring. In atherosclerosis, PET, mostly using ^18^F-fluorodeoxyglucose (^18^FDG), has been widely used to measure vascular inflammation, and the signal largely reflects inflammatory cell activity within plaques (32).

For myocardial damage, hybrid imaging approaches have shown that inflammatory cell recruitment can extend beyond the infarction site to the distant myocardium, supporting the concept of spatially distributed post-infarction inflammation (33). Longitudinal PET studies have further correlated myocardial inflammatory signals with post-MI remodeling trajectories (10).

### 4.2 USPIO/ferumoxitol-enhanced CMR for cellular inflammation

Cardiac magnetic resonance (CMR) can provide detailed spatial information about myocardial damage (edema, scarring) and, when paired with iron oxide nanoparticles, can also examine cellular inflammation related to macrophage activity. In patients with acute MI, ultra-small particles of iron oxide (USPIO) uptake have been shown in the infarcted and remote myocardium (34, 35). USPIO-enhanced CMR has been used to vascular and myocardial inflammation. (36, 37). Ferumoxytol-enhanced CMR has also been applied to detect myocardial macrophage activity within the first two weeks following acute MI (38).

For myocarditis, ferumoxytol/USPIO-enhanced CMR has been studied with mixed incremental value compared to conventional CMR sequences. This is informative for understanding when cellular tracers add discriminative power and when they do not (39, 40).

**Table 3** presents a comparative overview of methodological approaches to characterizing immune dynamics in cardiovascular disease across space and time. For each approach, the table details its spatial resolution capabilities, temporal applicability for serial measurements, key strengths, major limitations, and optimal applications within the spatiotemporal framework. This methodological toolset supports researchers and clinicians to select appropriate measurement strategies based on specific research questions and clinical contexts.

**Table 3:** Methodological approaches to unraveling spatiotemporal immune dynamics.

Modality	Spatial resolution	Temporal feasibility	Strengths	Limitations	Best use in framework
PET imaging (FDG, immune-targeted tracers)	Organ/regional	Serial longitudinal	Non-invasive; measures inflammatory load	Limited cellular specificity; radiation exposure	Monitoring intravascular and myocardial inflammation over time
CMR (including USPIO/ferumoxytol enhanced)	Tissue level	Serial (days–weeks)	Detects macrophage activity; scar characterization	Limited availability; contrast agent considerations	Infarct core and border zone inflammation
Single-cell RNA sequencing	Single-cell	Cross-sectional (tissue-dependent)	High-resolution immune phenotyping	Loss of spatial context	Defining phase-specific immune cell programs
Spatial transcriptomics/multi-omics	Near-cellular spatial mapping	Cross-sectional (limited to serial biopsy)	Preserves anatomical localization	Necessity of tissue access	Mapping compartment-specific immune niches
Serial plasma biomarker profiling	Systemic	Highly feasible	Minimally invasive; longitudinal	Limited compartment specificity	Tracking immune trajectories
Machine learning integration	Multimodal	Longitudinal-capable	Pattern detection; predictive modeling	Requires validation; interpretability challenges	Integrating phase + compartment data into predictive trajectories

### 4.3 Single-cell and spatial transcriptomics

Single-cell RNA sequencing (scRNA-seq) has enabled high-resolution characterization of immune and stromal heterogeneity in the human heart (14). Spatial multi-omics has extended this while preserving the anatomical context; in particular, human MI tissue mapping has revealed distinct spatial domains of immune activation and extracellular matrix remodeling (9). These approaches directly support the “partitioning” proposition of a spatiotemporal framework. Heart-focused methodological syntheses outline platform choices, analytical pitfalls, and best practices for translating spatial transcriptomics into human cardiovascular immunology (41). Single-cell and spatial approaches are increasingly considered complementary, linking cellular states to microanatomical niches throughout cardiovascular development and disease (42).

### 4.4 Serial biomarker profiling and kinetic modeling

Circulating biomarkers remain the most readily accessible window into cardiovascular inflammation, but single measurements obscure the kinetics. Serial sampling allows for the reconstruction of inflammatory trajectories (e.g., early and delayed peaks), which can be better correlated with remodeling risk than isolated time points. Phase-specific biomarker patterns can also be integrated with imaging readouts to infer compartmentalized sources and transition points (1, 2).

### 4.5 Computational integration and machine learning

**Figure 2:**
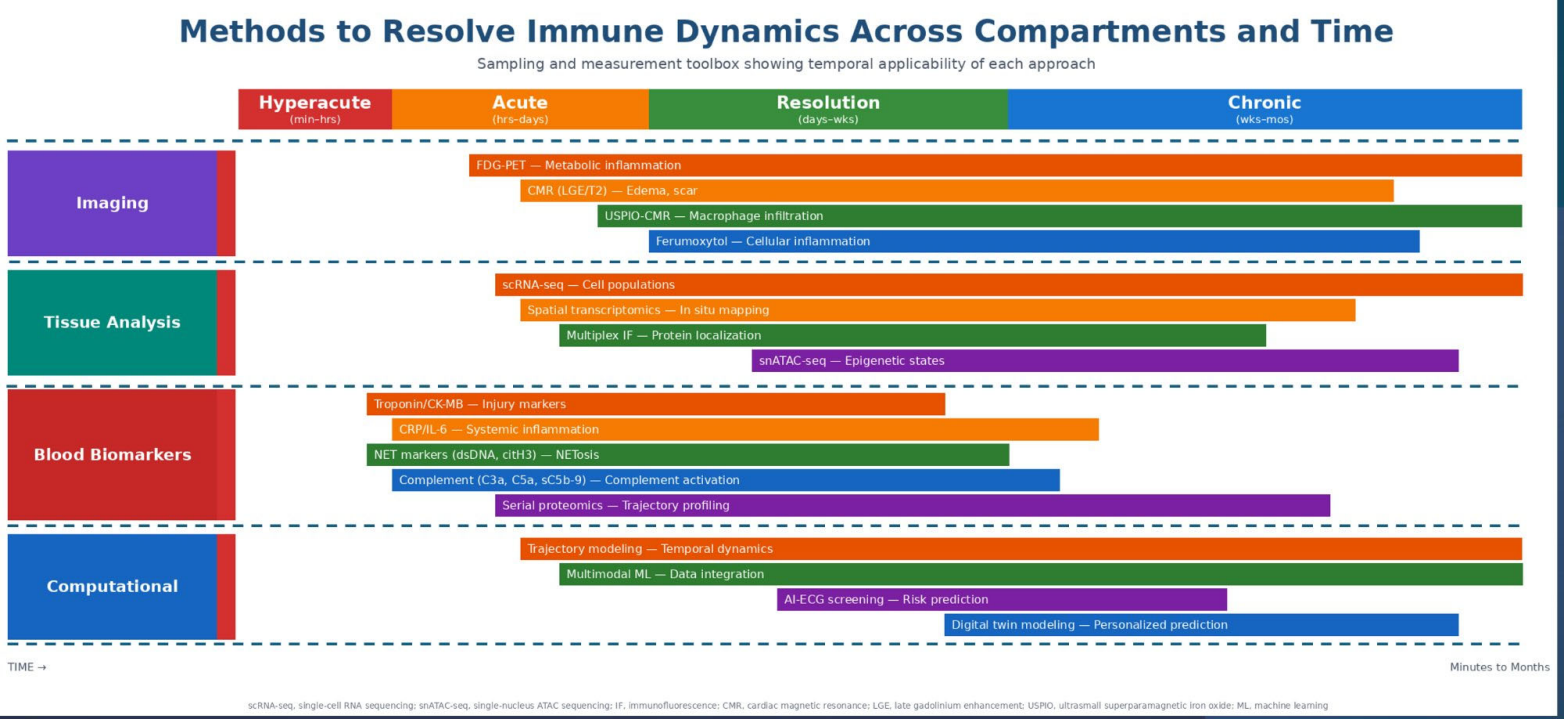
Phases and methods for unraveling immune dynamics over time. The figure is organized into four methodological domains: **(A)** Imaging modalities including FDG-PET, late gadolinium contrast-enhanced CMR and USPIO/ferumoxitol-enhanced CMR for T2 mapping and macrophage imaging; **(B)** Tissue analysis techniques including single-cell RNA sequencing (scRNA-seq), spatial transcriptomics, multiplex immunofluorescence, and single-nucleus ATAC sequencing (snATAC-seq); **(C)** Blood biomarker approaches including injury markers (troponin, CK-MB), inflammatory markers (CRP, IL-6), NET-related markers (extracellular DNA, citrullinated histone H3), complement activation products (C3a, C5a, sC5b-9), and serial proteomics; and **(D)** Computational approaches including trajectory modeling, multimodal machine learning, AI-assisted ECG scanning, and digital twin modeling. The bar length indicates the temporal window in which each method provides the most relevant information. AI, artificial intelligence; CMR, cardiac magnetic resonance; FDG, fluorodeoxyglucose; IF, immunofluorescence; LGE, late gadolinium enhancement; ML, machine learning; PET, positron emission tomography; scRNA-seq, single-cell RNA sequencing; snATAC-seq, single-nucleus assay for transposable-accessible chromatin sequencing; USPIO, ultra-small particles of iron oxide.

As multimodal datasets (imaging + omics + serial biomarkers) expand, computational integration becomes essential. Machine learning approaches can identify hidden phenotypes and predictive patterns in high-dimensional cardiovascular datasets (15, 43). In a spatiotemporal immunity framework, these tools are best positioned as integrators linking location-specific signals and time-dependent trajectories, rather than replacing mechanistic biology. In interventional device contexts, AI has been proposed as a way to integrate imaging, procedural variables, and device-patient factors to optimize device design and selection (44). These approaches integrate heterogeneous data to reconstruct immune trajectories. The temporal applicability and complementary strengths of these approaches are summarized in **Figure 2**.

## 5 NETs as a spatiotemporal case study

Neutrophil extracellular traps (NETs) offer a paradigmatic example of why cardiovascular immunity should be interpreted from a spatiotemporal perspective. NETs are chromatin-based structures adorned with granular and nuclear proteins that can enhance thrombosis, spread inflammation, and modulate interaction between immune cells. In cardiovascular events, NET biology is largely dependent on where NETs form (e.g., coronary thrombus vs. infarct microcirculation vs. periinfarct tissue) and when they are dominant (hyperacute occlusion vs. reperfusion vs. repair/remodeling). NETs illustrate how a single effector mechanism can be protective or harmful depending on the context (5, 17).

### 5.1 Early release, localization, and persistence

During hyperacute coronary occlusion, NETs are enriched at the culprit lesion site and within coronary thrombi. In patients with STEMI, neutrophils from the responsible lesion indicate NET formation, and coronary NET burden is associated with larger infarct size and worse ST segment resolution (18). Mechanistically, platelet-neutrophil interactions at the plaque rupture site can trigger NETosis and transmit prothrombotic signals. In the artery responsible for acute myocardial infarction, thrombin-activated platelets interacting with neutrophils have been associated with NET formation and the delivery of activated tissue factor, supporting *in-situ* amplification of NET-driven thrombogenicity (45).

NET biology is not restricted to the epicardial thrombus. Experimental ischemia-reperfusion models have demonstrated the presence of NETs within the myocardium and revealed that NET-mediated microthrombosis plays a role in the “blood flow arrest” phenomenon. A DNase-based reperfusion strategy (combination of DNase I with thrombolysis) improved myocardial perfusion and functional outcomes in a rat model, consistent with the concept that NET structures contribute to microvascular obstruction after reperfusion (46).

At the systemic level, circulating markers associated with NET formation, such as dsDNA and citrullinated histone H3, have been associated with infarct size and adverse remodeling in STEMI cohorts, supporting the idea that NET-related signals persist beyond the earliest hours (47). These foundlings demonstrate that NETs occurs across a continuum from thrombus to myocardial microvasculature, and systemic circulation.

### 5.2 Site-specific effects: prothrombotic and tissue-modulating biology

Spatial compartmentalization is central to the interpretation of NET effects. In the intravascular compartment, NETs promote immunothrombosis through scaffolding of platelets and clotting factors and delivery of prothrombotic mediators (17, 45). In the microcirculation and infarct core, NET-mediated microthrombosis can contribute to impaired reperfusion and extension of damage, consistent with experimental reperfusion findings and “no reflow” pathobiology (46). In contrast, in the evolving tissue repair environment, NETs can also influence macrophage polarization and immune resolution programs, pointing to potentially different roles across phases and compartments. Notably, genetically disrupting NET formation does not always improve outcomes. In a mouse MI model, PAD4 deficiency (a key enzyme involved in chromatin decondensation during NET formation) was associated with exacerbation of acute inflammation and increased early tissue damage; mechanistic signals suggest NET-dependent support of macrophage polarization toward reparative phenotypes; however, subsequent functional effects are more complex (48). These findings highlight that suppression of NET formation may have some drawbacks, particularly when applied indiscriminately over time or without compartment-specific targeting.

### 5.3 Therapeutic window assessments and translational signals

Since NETs contribute to both thrombosis and inflammation, strategies targeting NETs are attractive; however, their success is likely dependent on precise timing. The hyperacute window (minutes–hours) during coronary occlusion and immediate reperfusion may be the optimal time for therapies aimed at reducing NET-mediated thrombosis and microvascular obstruction (18, 46). The recent cardiovascular-focused synthesis of NET formation pathways and therapeutic entry points further supports timing and site-specific NET modulation rather than uniform NET suppression (49). In contrast, later stages (days–weeks) may require approaches that modulate NET-derived inflammation while preserving repair processes.

Clinical translational evidence is emerging. In a randomized STEMI trial context, IL-6 receptor inhibition with tocilizumab was associated with reductions in NET markers and associations between NETs and myocardial damage; suggesting that modulation of upstream inflammatory signaling may influence NET biology in humans and contribute to myocardial rescue (50). While these findings do not identify NETs as the sole mediator of benefit, they strengthen the feasibility of targeting inflammatory networks that intersect with NET formation.

**Figure 3:**
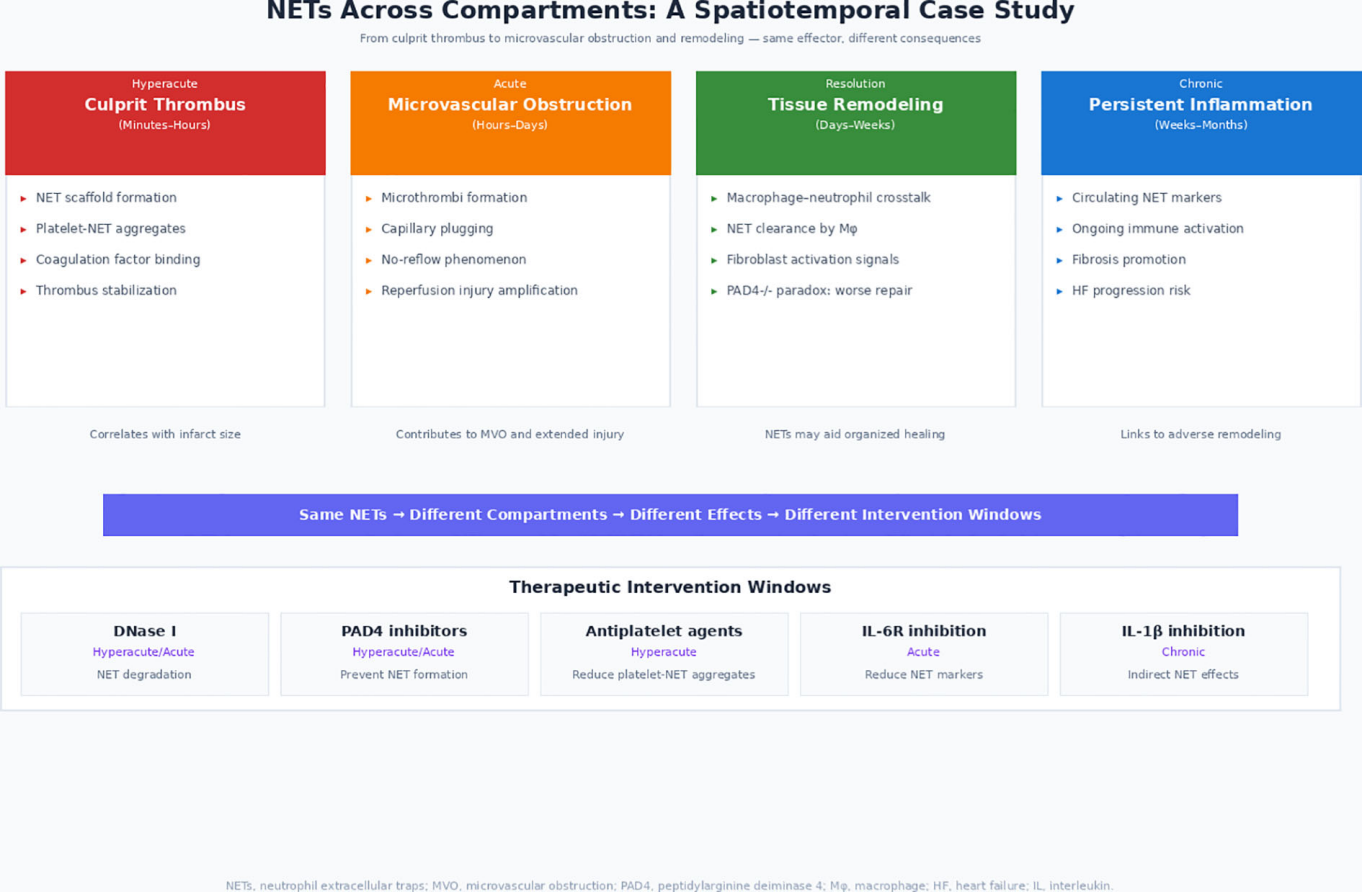
NETs across compartments: A spatiotemporal case study. This figure illustrates how neutrophil extracellular traps (NETs) exemplify the importance of temporal and spatial context in cardiovascular immunity. **(A)** Hyperacute phase (Culprit Thrombus): NETs provide a prothrombotic scaffold within the intravascular compartment, and the NET load is correlated with infarct size. Key processes include NET scaffold formation, platelet-NET aggregates, coagulation factor binding, and thrombus stabilization. **(B)** Acute phase (Microvascular Occlusion): NET-mediated microthrombi contribute to capillary occlusion, no-reflow phenomenon, and increased reperfusion injury in the microvasculature and infarct zone. **(C)** Resolution phase (Tissue Remodeling): NETs participate in macrophage-neutrophil interaction and fibroblast activation in the infarct core and border zone. The PAD4 deficiency paradox (worse repair in PAD4-/- models) suggests that NETs may contribute to organized recovery. **(D)** Chronic phase (Persistent Inflammation): Circulating NET markers are associated with persistent immune activation in the distant myocardium and systemic circulation, development of fibrosis, and adverse remodeling. The lower panel shows the therapeutic intervention windows for strategies targeting NETs: DNase I (NET degradation, hyperacute/acute), PAD4 inhibitors (prevention of NET formation, hyperacute/acute), antiplatelet agents (reduction of platelet-NET aggregates, hyperacute), IL-6 receptor inhibition (reduction of NET markers, acute), and IL-1β inhibition (indirect NET effects, chronic). HF, heart failure; IL, interleukin; Mφ, macrophage; MVO, microvascular obstruction; NETs, ​​neutrophil extracellular traps; PAD4, peptidylarginine deiminase 4; STEMI, ST-segment elevation myocardial infarction.

NETs exemplify the fundamental rationale for a spatiotemporal framework: the same effector construct can lead to thrombosis in one compartment, disrupt reperfusion in another, and subsequently influence repair programming; meaning that interventions require adaptation to both anatomical niche and phase-specific biology. This spatiotemporal complexity is schematically illustrated in **Figure 3**, using NETs as a representative example.

## 6 Clinical and therapeutic implications of a spatiotemporal framework

A spatiotemporal model of cardiovascular immunity has direct implications for therapeutic design, biomarker interpretation, and risk stratification. Anti-inflammatory therapies in cardiovascular disease often show heterogeneous or context-dependent outcomes. These mixed results may reflect mismatch between therapeutic timing, anatomical targeting, and immune-phase biology.

### 6.1 Timing-dependent intervention strategies

Clinical experience with inflammation-targeted therapy demonstrates the importance of timing. In the CANTOS study, IL-1β inhibition provided evidence in principle that targeted cytokine modulation could improve outcomes by reducing recurrent cardiovascular events in patients with prior myocardial infarction and elevated inflammatory markers (6). However, broad anti-inflammatory approaches in unselected populations have not consistently shown benefit.

Similarly, colchicine has reduced cardiovascular events in both recent myocardial infarction (COLCOT) and chronic coronary heart disease (LoDoCo2) populations, suggesting that suppression of innate immune pathways may be beneficial in both subacute and chronic phases (7, 8). However, the magnitude of the benefit and optimal treatment windows remain areas of investigation.

Beyond classic anti-inflammatory therapies, emerging cardiometabolic drugs may also exhibit immunomodulatory effects related to post-infarction remodeling. Sodium-glucose cotransporter-2 (SGLT2) inhibitors and glucagon-like peptide-1 receptor agonists (GLP-1RA), commonly used in heart failure and metabolic diseases, have been suggested to influence inflammatory pathways, oxidative stress, and immune cell activation. While their cardiovascular benefits are thought to involve multiple mechanisms, increasing evidence suggests that immunomodulation may contribute to their protective effects in heart failure populations (51, 52).

From a spatiotemporal perspective, hyperacute interventions may aim to reduce immunothrombosis and microvascular obstruction, while subacute strategies may focus on limiting excessive cytokine amplification. Later-phase interventions, in turn, may prioritize promoting resolution pathways or mitigating non-compliant fibrosis. Failure to distinguish these ranges risks blunting beneficial inflammatory programs or missing critical early factors of injury.

### 6.2 Spatial targeting: local and systemic immunomodulation

Anatomical segmentation introduces an additional therapeutic dimension. Systemic immunomodulation simultaneously targets multiple immune niches such as plaque, infarct core, border zone, distant myocardium, and circulating leukocyte reservoirs. However, pathological activity can be spatially concentrated.

For example, PET imaging studies show that persistent vascular inflammation predicts recurrent events in atherosclerosis, suggesting that persistent plaque-specific inflammation may necessitate targeted intervention (32). In myocardial infarction, inflammation-sensitive imaging has revealed regional heterogeneity between the infarcted and distant myocardium, demonstrating that localized immune activity may contribute to remodeling beyond the primary lesion (10).

Device-tissue interfaces offer an additional opportunity for spatially selective modulation. Biomaterial engineering approaches that reduce foreign body responses at the implant surface or modulate macrophage polarization can alleviate restenosis or fibrosis without requiring systemic immunosuppression (12). These strategies exemplify how anatomical niche-specific modulation can be incorporated into cardiovascular intervention design.

Cell-based regenerative therapies can also interact with the immune environment following myocardial infarction. Mesenchymal stem cells have demonstrated immunomodulatory properties, including suppression of pro-inflammatory cytokine signaling and support of reparative macrophage phenotypes. Similarly, cardiomyocytes derived from induced pluripotent stem cells may contribute to myocardial repair via paracrine immune modulation and angiogenic signaling (53, 54). Recent experimental studies also suggest that bioengineered epicardial patches can trigger macrophage-mediated reparative pathways in chronically infarcted myocardium (55).

### 6.3 Biomarker-guided personalization

Circulating inflammatory biomarkers such as CRP and IL-6 indicate residual inflammatory risk (6). However, interpretation of such biomarkers without temporal and spatial context may be incomplete.

Serial biomarker assessment can identify patients with persistent inflammatory trajectories following acute MI and potentially differentiate those remaining in a prolonged inflammatory phase from those who have undergone appropriate resolution. Integrating biomarker kinetics with imaging-derived spatial data can enable the classification of patients into spatiotemporal immune phenotypes.

This approach is consistent with emerging precision medicine paradigms in cardiology where multimodal risk assessment is used to improve therapeutic selection (43).

### 6.4 Risk classification based on immune trajectory

Beyond short-term event reduction, a spatiotemporal immune model can improve long-term risk prediction. Ongoing inflammatory activation after myocardial infarction has been associated with adverse ventricular remodeling and progression of heart failure (2). Identifying patients with persistent cytokine elevation, inflammation detected by ongoing imaging, or non-resolving immune trajectories defined by transcriptomic signatures may allow for earlier intervention.

Patient heterogeneity can further alter immune processes following myocardial infarction. Age-related immune remodeling, sex-dependent inflammatory responses, and concomitant metabolic diseases can alter innate and adaptive immune activation after injury (56, 57). Additionally, concomitant immunosuppressive therapies and baseline immune conditioning can influence biomarker profiles and treatment response. These variables may partially explain why inflammatory biomarkers and treatment effects differ across patient populations. Including demographic and clinical modifiers in temporal and spatial immune models can improve risk stratification and support more individualized immunomodulatory strategies.

Notably, risk stratification should move beyond single biomarker thresholds towards trajectory-based assessment. Trajectory-based stratification can be improved by incorporating myeloid subset heterogeneity (e.g., CCR2-driven inflammatory recruitment programs), which is increasingly emphasized in vascular inflammatory disorders such as aortic aneurysm biology (58, 59). For example, a declining inflammation curve during the first week after a myocardial infarction may indicate adequate resolution, while a secondary rise or sustained plateau may signal ongoing tissue damage or impaired efferocytosis.

**Figure 4:**
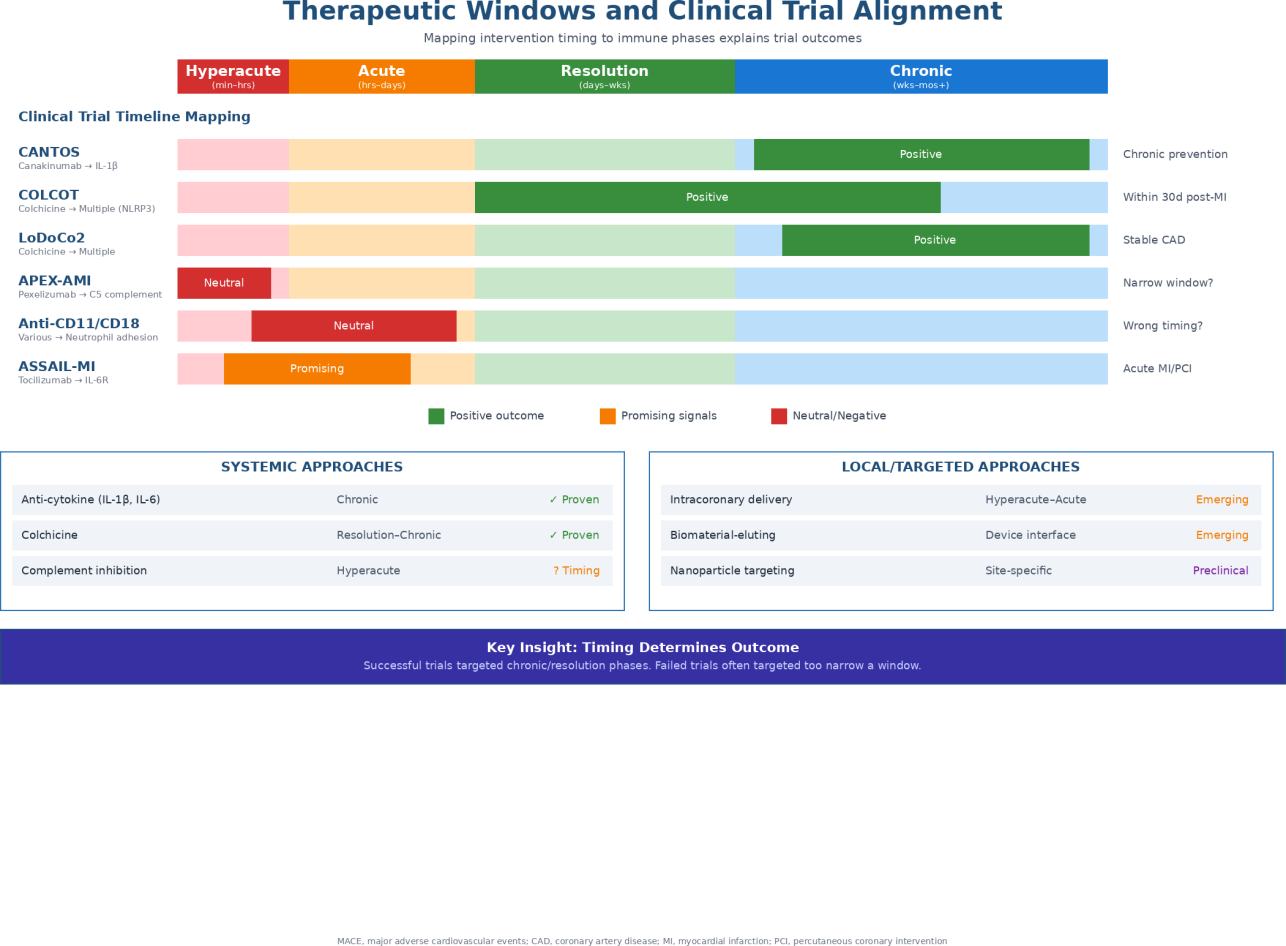
Therapeutic windows and clinical trial alignment. This figure shows how the timing of intervention affects therapeutic outcomes by mapping immunomodulatory clinical trials according to the temporal phases of cardiovascular immunity. Upper panel (clinical trial timeline mapping): Colored bars show the temporal window targeted by each trial. Successful trials with favorable outcomes (green) include CANTOS (canakinumab targeting IL-1β in chronic secondary prevention), COLCOT (colchicine initiated within 30 days after myocardial infarction), and LoDoCo2 (colchicine in stable coronary artery disease). ASSAIL-MI (tocilizumab targeting the IL-6 receptor in acute MI) shows promising signals (yellow). Studies yielding neutral results (red) include APEX-AMI (pexelizumab targeting C5 complement, potentially a very narrow window) and anti-CD11/CD18 studies (targeting neutrophil adhesion, potentially mistimed or mistargeted). Lower panels (treatment strategies): Systemic approaches with proven efficacy include anti-cytokine therapy (IL-1β, IL-6) in the chronic phase and colchicine from remission to chronic phases; complement inhibition in the hyperacute phase is under investigation for optimal timing. Local/targeted approaches such as intracoronary delivery, biomaterial-delivering devices, and nanoparticle targeting represent emerging or preclinical strategies. Key insight: Successful studies have mostly targeted the chronic or remission phases with continuous intervention, while studies yielding neutral results have generally used a very narrow intervention window or targeted inappropriate phases. CAD, coronary artery disease; CV, cardiovascular; IL, interleukin; MACE, major adverse cardiovascular events; MI, myocardial infarction; NLRP3, NOD-like receptor protein 3; PCI, Percutaneous coronary intervention.

Spatiotemporal integration transforms immune markers from static indicators into dynamic predictors of structural and functional remodeling. The relationship between immune phase, therapeutic timing, and clinical trial outcomes is summarized in **Figure 4**.

## 7 Future trends: towards real-time, precise cardiovascular immunology

Advances in spatial, molecular, and computational tools enables transition from retrospective characterization to trajectory-driven immune management. A spatiotemporal framework reorganizes current knowledge and make possible new experimental designs and therapeutic strategies.

### 7.1 Real-time immuno-monitoring technologies

Future progress depends on the ability to monitor immune activity with longitudinal and anatomical specificity. Advances in molecular imaging, including immune-targeted PET tracers and macrophage-sensitive MRI techniques, have already demonstrated applicability for monitoring *in vivo* inflammatory load (10, 32). Extending tracer specificity to defined immune subsets or activation states could allow for differentiation between destructive inflammation and reparative immune activity.

At the tissue level, spatial transcriptomics and multi-imaging technologies are rapidly advancing in terms of resolution and efficiency. Human myocardial atlases have begun to define the basic cellular architecture (14), and spatial multi-omics mapping of infarcted myocardium has revealed regionally distinct immune programs (9). Where clinically feasible, the integration of serial tissue sampling with imaging and blood biomarkers can support the dynamic reconstruction of evolving immune niches.

Emerging high-dimensional plasma profiling and proteomics are further expanding the capacity for longitudinal immune monitoring. Multi-protein inflammatory panels have demonstrated the ability to classify cardiovascular risk beyond single biomarkers, suggesting that composite signatures may better reflect complex immune states (6).

### 7.2 AI-powered immune trajectory modeling

As data complexity increases, computational modeling to integrate spatiotemporal signals will become essential. Machine learning approaches have already demonstrated the capacity to extract predictive cardiovascular patterns from imaging, electrocardiography, and electronic health records (15, 43). Extending these approaches to immunological datasets offers the possibility of identifying latent immune phenotypes corresponding to different remodeling trajectories.

Future models could incorporate temporal slopes, regional imaging features, and molecular signatures to predict whether a patient is transitioning toward adaptive repair or maladaptive remodeling, rather than relying on static thresholds (e.g., a single CRP value). Such models must remain biologically interpretable to avoid opaque predictions detached from mechanistic insight.

The integration of multimodal immunomodulation data into predictive architectures is consistent with broader initiatives in precision cardiovascular medicine where risk prediction increasingly involves dynamic and multivariate signals. In interventional workflows, such models can ultimately support personalized device planning and immune-informed risk prediction by integrating imaging, procedural variables, and device-patient factors (44).

### 7.3 Precise immunomodulation

A spatiotemporal framework naturally leads to the concept of precise immunomodulation. Instead of uniform systemic suppression, treatments can be matched according to phase (hyperacute thrombo-inflammatory, acute inflammatory, resolution, chronic remodeling), site (intravascular, infarct core, border region, distant myocardium, device-tissue interface), or immune phenotype (neutrophil-dominant, monocyte-driven, complement-amplified, fibrotic transition).

For example, complement-targeted therapies may be most sensible in early reperfusion windows where complement-mediated microvascular damage is prominent (20), whereas interventions that enhance macrophage-mediated resolution may be prioritized in the repair phase (2). Device-related inflammation may require biomaterial-specific surface modifications instead of systemic immunosuppression (12). Clinical trials may increasingly adopt adaptive designs that include biomarker-guided enrollment or stratification according to the course of inflammation. Lessons from IL-1β inhibition (6) and colchicine studies (7, 8) show that therapeutic success depends not only on pathway choice but also on identifying patients at risk of residual inflammation.

### 7.4 Standardization of spatiotemporal reporting in cardiovascular research

A crucial next step for the field is methodological standardization. Cardiovascular inflammation studies often report single time points or lack clear anatomical resolution. Inclusion of phase and compartment standard descriptors in preclinical and clinical trials will facilitate inter-study comparison and improve translational agreement. Future reporting frameworks may require explicit definition of the following:

- Time elapsed since injury/intervention.
- Tissue or anatomical region analyzed.
- Dominant immune cell populations and activation states.
- Concurrent systemic inflammatory markers.

Such standardization will enable more consistent synthesis of data and more precise mapping of treatment intervals.

### 7.5 Limitations

Several limitations need to be acknowledged. First, the proposed spatiotemporal framework represents a conceptual synthesis of the existing literature rather than a quantitative meta-analysis. Second, many of the mechanistic insights derive from experimental models of myocardial infarction, and their direct translation to human disease may differ. Third, current imaging and spatial transcriptomic technologies remain limited in terms of accessibility and temporal resolution, restricting their routine clinical application. Finally, immune responses following myocardial injury are affected by significant patient heterogeneity, including age, concomitant diseases, and pharmacological exposures, which may alter the trajectories described here.

## 8 Conclusion

Cardiovascular immunity is neither temporally linear nor spatially homogeneous. Rather, it represents a dynamic, multi-compartmental process developing in distinct but overlapping phases, from hyperacute thrombo-inflammatory activation to chronic remodeling. Traditional definitions of cardiovascular inflammation have emphasized molecular mediators and cellular subsets in isolation. However, evidence from imaging, spatial transcriptomics, clinical trials, and systems biology demonstrates that immune processes should be interpreted within the context of both anatomical niche and temporal phase.

By integrating temporal immune phases with spatial compartmentalization, including the intravascular environment, infarct or lesion core, peri-infarct border zone, distant myocardium, and device-tissue interface, we propose a four-dimensional framework for mapping cardiovascular immunity dynamics. This architecture clarifies why the same pathways can have different effects depending on the phase, why anti-inflammatory therapies yield heterogeneous results, and why biomarker interpretation requires contextual alignment. This also underscores the need to distinguish protective clearance responses from the maladaptive persistence of inflammation and fibrosis.

This framework is not merely conceptual. Advances in molecular imaging, single-cell and spatial omics, serial biomarker profiling, and computational modeling are enabling the reconstruction of immune trajectories with increased resolution. Integrating these methods creates the possibility of trajectory-focused intervention by matching treatment to phase, region, and immune phenotype. By doing so, cardiovascular immunology can shift from pathway suppression to precise immunomodulation.

Future research should standardize reporting of immune phase and anatomical region, longitudinal assessment of inflammatory trajectories, and adaptive trial designs incorporating biomarker-focused stratification. As cardiovascular disease remains the leading cause of global mortality, improving immunological frameworks is not only a scientific opportunity but also a clinical imperative. A spatiotemporal approach transforms cardiovascular inflammation from a static risk factor into a dynamic, measurable, and potentially modifiable process. Mapping this immune environment with four-dimensional resolution could ultimately bridge the gap between mechanistic understanding and personalized cardiovascular care.
